# A Systematic Review of the Impact of Placement Instability on Emotional and Behavioural Outcomes Among Children in Foster Care

**DOI:** 10.1007/s40653-023-00606-1

**Published:** 2024-02-28

**Authors:** Darren Maguire, Keziah May, David McCormack, Tim Fosker

**Affiliations:** Queen’s University, Belfast, Northern Ireland

**Keywords:** Foster care, Placement Instability, Care-related Factors, Mental Health, Behaviour Problems, Systematic Review

## Abstract

Foster care children are a highly vulnerable population and their experiences in care are considered crucial to their developmental and psychosocial wellbeing. Placement instability has been considered a possible risk factor for developmental difficulties due to its impact on the development of a reparative attachment relationship and sense of relational permanence. The current review synthesises the literature regarding the impact of placement instability on behavioural and mental health outcomes in foster care children. Three major databases and grey literature sources were searched for all relevant quantitative research published by July 2019. Titles and abstracts of 2419 articles were screened following searches, with full texts obtained for 51 studies and 14 included in the final review. All were subject to quality assessment by two independent reviewers. Results indicated that placement instability was a consistent predictor of externalising behaviour in children, although some evidence was counter-indicative in this regard. There was also evidence to suggest a relationship with internalising behaviours, and mental health difficulties, in particular PTSD symptoms. Methodological quality and design varied between studies which limited direct comparisons. Most notably, there was a lack of consensus on how to quantify and measure placement instability and many studies failed to control for potentially confounding care-related variables. The review highlights that instability seems to result in negative psychological outcomes, although the extent of this relationship remains unclear. The review’s findings are discussed with reference to research and clinical implications.

The number of children removed from the care of their parents is increasing and these children will often enter the foster care system for a period (Rice et al., 2017). Children in care have had significant negative life experiences that often result in developmental cognitive, emotional and behavioural difficulties beyond the frequency experienced by the typically developing child population (Chambers et al., 2010; Havlicek et al., 2013). Developmental delay (Sawyer et al., 2007), sexually inappropriate behaviour (Prentky et al., 2014), substance misuse (Gabrielli et al., 2016), and emotional and behavioural difficulties (Tarren-Sweeney, 2008) have all been reported. In a recent systematic review, Oswald et al. (2010) extrapolated that 36–61% of the foster care population have behavioural problems within the clinical range, with 32–44% meeting criteria for diagnosis of a psychiatric disorder. Long-term outcomes into adulthood are also poor, with a recent study of over thirty thousand young people in out of home care in Finland being 1.4 to 5 times more likely to experience adverse outcomes upon leaving care (Sariaslan et al., 2022).

Nevertheless, the goal of looked after children’s services is placement permanency with the view to reducing future negative life experiences and improving life outcomes (Bell & Romano, 2017). This is not always possible, and children often enter placements of varying lengths, or varying stability. Placement instability, generally defined as several changes in residency and/or caregiver for a child following entry into care, is a potential predictor of negative developmental outcomes (Garcia et al., 2015; McGuire et al., 2018; cf. Table 1 for variation in the specific definition). A recent review of children in care in the USA suggested that 25% experience at least one disruption of placement in their first 18 months (Dolan et al., 2013) and a third of children in care in Australia have 5 or more changes in placement over the course of their childhood (Rice et al., 2017). A recent UK study reported that 43% of children in care will find a settled placement early in their care experience and remain there until their 18th birthday, which the authors suggest represents high levels of stability (McSherry & Fargas-Malet, 2018). There are a variety of reasons why a child’s placement may break down, such as the child’s age, behavioural difficulties, placement type, placement quality and changes in the foster carer’s situation (see Konijn et al., 2019 for a review). Whatever the reason, these placement breakdowns can be traumatic for the child and represent the loss of another attachment figure following removal from their birth parents (Kernreiter et al., 2020).

**Table 1:** Variations of placement instability definition between studies reviewed

Definition of placement instability	Authors (year)
Any change of residence that constitutes care by an alternative caregiver, including foster, kinship or residential placements, juvenile detention or inpatient care	Aarons et al. (2010) Herrenkohl et al. (2003) Prentky et al. (2014) Ryan and Testa (2005) Zima et al. (2000)
Any change of residence that constitutes care by an alternative caregiver, including foster, kinship or residential placements, juvenile detention or inpatient care and also includes changes of school placement	Bederian-Gardner et al. (2018)
Any change of residence that constitutes care by an alternative caregiver. Entry into a “receiving centre” between placements also counts as 1 placement change	Newton et al. (2000)
The occurrence of at least 1 placement change between time points	Barber and Delfabbro (2003) Proctor et al. (2010) Rubin et al. (2007)
Whether the child was subject to 1 or more than 1 placement over the course of the study	Lewis et al. (2007)
The average number of placements per year in care, including foster, kinship and residential placements, “other”	Okpych and Courtney (2018)
Ratio of time in present placement/time in care	Tarren-Sweeney (2008)
Continuous variable for all placements for first 18 months following entry into care, then categorical variable based on whether or not placement was the same at 6 month intervals	Villodas et al. (2016)

The results of a number of research studies suggest that there may be a relationship between placement instability and emotional and behavioural outcomes (Bederian-Gardner et al., 2018; Proctor et al., 2010). Placement instability may correspond with frequent changes in school, environment, and peer relationships, all of which may exacerbate children’s behaviour difficulties, result in reduced wellbeing, and greater levels of health service utilisation (Konijn et al., 2019; McGuire et al., 2018; Rubin et al., 2007). Despite increased levels of health service utilisation, the continuity of care can be disrupted by more frequent changes of residence, and thus children in this position may not have their care needs met (DeGiuseppe & Christakis, 2003).

A large proportion of research has focused on the impact of child emotional and behavioural needs on the subsequent breakdown of foster placements (e.g. James, Landsverk & Slymen, 2004; Chamberlain et al., 2006; also see Rock et al., 2015, for a review). Reviews of the literature by Rock et al. (2015) and Konijn et al. (2019), while focusing on the impact of emotional and behavioural difficulties on placement stability, also note the potential interactive relationship between placement instability and emotional and behavioural outcomes. The addition of studies showing a predictive relationship between instability and emotional and behavioural outcomes are clearly suggestive of there being a bidirectional relationship. In order to redress the imbalance of systematic reviews focusing on the impact of emotional and behavioural difficulties on placement instability, this review will explore emotional and behavioural difficulty as an outcome of placement instability as a companion to previous systematic reviews focusing on placement instability outcomes (i.e. Rock et al., 2015, Konijn et al., 2019).

## Method

This review was conducted following the Preferred Reporting Items for Systematic Reviews and Meta-Analyses (PRISMA) statement (Moher et al., 2009). All literature searches and later quality assessments were conducted by two independent reviewers (DM and KM). Reviewers explored the literature independently, collated the information, discussed differences of opinion, and reached consensus based on the inclusion and exclusion criteria for studies. Two further members of the research team (DMcC and TF) were available to resolve disagreements as required.

The search strategy was based on guidance published by Petticrew and Roberts (2006) for best practice in systematic reviews of quantitative social care research (see also Stoll et al., 2018).

This review protocol was pre-registered on the systematic review register PROSPERO in August 2019 (ID: CRD42019149011).

### Search Strategy

A literature search of the following databases was conducted in July 2019:

- PsycINFO (1806-present)
- Scopus
- Web of Science
- Sources of grey literature
  - OpenGrey
  - ProQuest Digital Dissertations

Literature searches were conducted independently by two members of the review team (DM and KM), using the same search terms and databases. The review employed the following search terms (“foster care” OR “foster children” OR “Looked After Children” OR “LAC”) AND (“placement instability” OR “placement stability” OR “placement breakdown” OR “drift” OR “multiple placements” OR “caregiver stability” OR “caregiver instability” OR “instability” OR “stability”). These search terms were generated from clinical knowledge as suitably inclusive and were based on comparable literature reviews exploring outcomes in foster care children (e.g. O’Higgins, Sebba & Gardner, 2017; Rock et al., 2015). The review had two inclusion criteria which resulted in the exclusion of studies which used solely qualitative methods, and research published only in book chapters or presentations:

1. Quantitative or mixed-methods research on the impact of placement instability on emotional and behavioural outcomes in children in foster care.
2. Full text articles published in the English language.

**Fig. 1:**
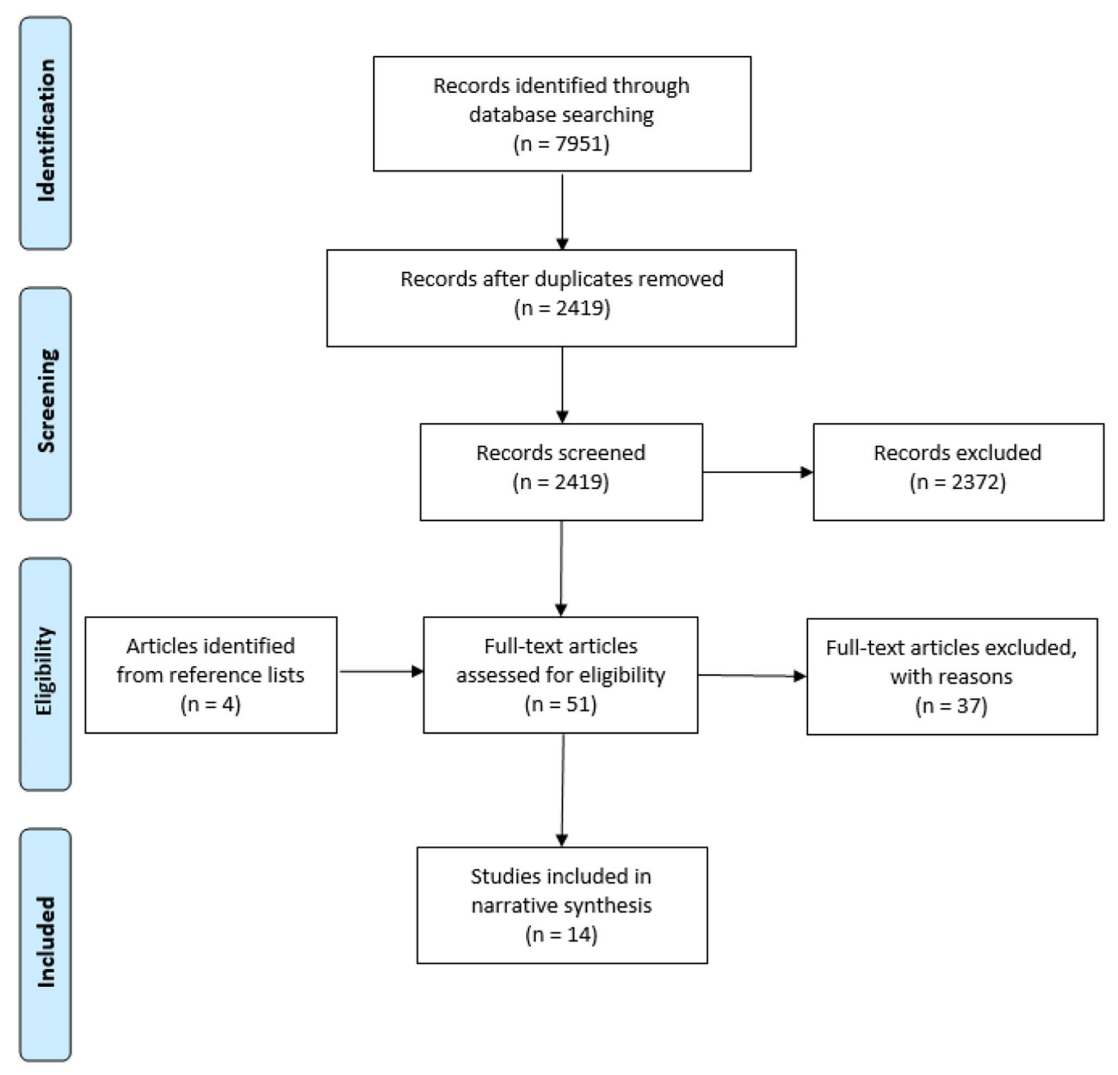
PRISMA Flow Chart (Moher et al., 2009)

The above search terms initially identified 7951 publications catalogued using EndNote™. Duplicates were removed, leaving 2419 to be screened. The above inclusion criteria were applied to each publication title. Publication abstracts were reviewed where required to establish meeting the inclusion criteria. This process yielded 47 articles which were felt to be potentially suitable for inclusion. These articles were then subject to a full review, conducted by reading the body of the text and confirming against the inclusion criteria again. Backward citation searching was employed for these 47 articles, which generated a further four articles. Thirty-seven of these articles were rejected from the final synthesis as they did not meet the inclusion criteria. The main reasons for rejection were that the studies explored placement instability as an outcome of emotional and behavioural difficulties (n = 18), used a sample including adults over the age of 18 (usually care leavers, n = 7), or employed a solely qualitative methodology (n = 4). There was disagreement regarding inclusion of one paper between the two reviewers. The paper in question was eventually included in the review following discussion with reference to the inclusion criteria. Fourteen studies were therefore selected for inclusion in the current review (see Fig. 1 for flowchart detailing the article selection process).

### Quality Assessment

The studies included in the review were subject to methodological quality assessment by two review authors (DM and KM), with additional authors (DMC and TF) available to resolve disagreements by discussion and consensus. As the studies in this review employed quantitative methods, the Quality Assessment Tool for Observational, Cohort and Cross-Sectional Studies (NIH, 2014) was used to assess study quality and risk of bias. The tool enabled a rating of “good”, “fair” or “poor” quality rating for each study included in the review. Both the criteria of the tool and the reviewers’ own judgement were used to highlight the relative strengths and weaknesses of each study.

Further detailed examination of each study was also conducted to assess for additional sources of bias, as recommended by Petticrew and Roberts (2006). QA ratings were assessed individually, then pooled together and evaluated for differences of opinion. Initial ratings indicated 93.30% agreement between raters (κ = 0.860, *p* < .001). Any differences of opinion were discussed, and consensus was subsequently reached on all items.

### Data Synthesis

While the papers in question all represented studies with relevant information to the review, there was also considerable heterogeneity between them. No papers included randomised samples, with most being cross-sectional analyses or longitudinal cohort studies. Four papers did not include standardised effect sizes, these were ones which incorporated analysis of case files or used semi-structured interviews. There were also discrepancies in relation to the directionality of the studies, with three papers exploring behavioural wellbeing instead of difficulty. There was substantial clinical diversity between samples, with notable differences in age range, study focus and the measures used. Consequently, it was decided that the current review would make best use of the data by conducting a narrative synthesis. This allows for ostensibly diverse methodologies and populations to be more directly compared while also exploring different contexts and mediating factors between them and a more nuanced exploration of strengths and flaws of the literature base (Campbell et al., 2017). A narrative synthesis also allows for the differences between studies to be highlighted in a manner which may contribute to future researchers refining this work in subsequent studies (Popay et al., 2006). Narrative synthesis is the recommended review methodology when exploring literature of this type (Ryan, 2013).

## Results

### Description of the Included Studies

Quality Assessment ratings indicated that seven studies were considered to have “Good” methodological designs and seven studies had “Fair” designs. No studies were rated as poor. Of the 14 studies included in the review, five used a cross-sectional design, while nine studies were longitudinal. Longitudinal study length was highly variable, ranging from eight months (Barber & Delfabbro, 2003) to 14 years (Herrenkohl et al., 2003).

Nine of the 14 studies included data from larger prospective cohort studies. Two of these studies used participants sampled from the same wider cohort (Aarons et al., 2010; Rubin et al., 2007) and a separate two studies drew from a different cohort (Proctor et al., 2010; Villodas et al., 2016). Only 12 of the 14 studies can therefore be assumed to have independent samples.

Sample sizes varied considerably between studies, ranging from 102 participants (Lewis et al., 2007) to 4085 participants (Ryan & Testa, 2005; median = 338, interquartile range = 367). There was considerable heterogeneity between the ages of participants in each study (overall range 0–18 years old). Six studies involved a child sample (i.e. less than 12 years old), five employed an adolescent sample (12 years and older) and four studies included both child and adolescent participants. One study included male participants only (Prentky et al., 2014). Ten studies reported an even gender split. A further three studies had a disproportionately female sample (Herrenkohl et al., 2003; Lewis et al., 2007; Okpych & Courtney, 2018) and one sample was predominantly male (Bederian-Gardner et al., 2018). Twelve of the 14 samples were generated from a population in the USA, with two remaining studies emanating from Australia (Barber & Delfabbro, 2003, Tarren-Sweeney, 2008).

Of note, there was considerable variation in the definition of placement instability between included studies. For example, several papers chose to include all changes of residence, regardless of length of time, and included a range of different potential placements e.g. foster care, residential placements or inpatient care. Others chose to also include respite placements and school moves in their total. Finally, some chose to define instability as a categorical variable based on whether the child remained in a stable placement between defined time points, not accounting for number of moves in that time (e.g. Proctor et al., 2010; see Table 1).

The focus of three of the studies was on the mental health of children in foster care in the context of placement instability (Bederian-Gardner et al., 2018, Okpych et al., 2018, Tarren-Sweeney, 2008), with the remaining 11 studies focusing on the children’s behavioural wellbeing. Two studies recorded placement instability by asking participants to recount placement changes (Bederian-Gardner et al., 2018; Herrenkohl et al., 2003) with all others using a form of case note analysis. Eleven studies used self-report or carer-report questionnaires to explore emotional and behavioural outcomes. Of these studies, eight used the Children’s Behaviour Checklist (CBCL; Achenbach, 2004), with a range of measures used for the other studies. Further details on individual studies are included in Table 2 in publication date order.

**Table 2:** Overview of Included Studies

Authors (date) & country	Design	Study Length	Sample Demographics	Groups	Measure(s)	Quality Appraisal Rating	Findings
Newton et al. (2000), USA	Prospective Cohort Study	12 months	N = 415 Age range = 2–17 years Gender − 47% male	Divided into “volatile” and “non-volatile” groups based on median split (median = 4) of placement changes	CBCL	Good	Children in volatile placement group (n = 98) had significantly greater internalising (e.g. social withdrawal, anxious behaviours) and externalising (rule breaking and aggressive behaviours) difficulties in comparison to non-volatile group, with small effect sizes.
Zima et al. (2000), USA	Cross-sectional	N/A	N = 302 Age range = 6–12 years Gender = 47% male	Acute vs. Chronic placement groups	Abbreviated CBCL	Fair	High levels of behaviour difficulties in foster care sample, but these difficulties were not associated with placement instability.
Barber & Delfabbro (2003), Australia	Prospective cohort study	8 months	N = 235 Age range = 4–17 years Gender = 51% male	3 placement groups, based on whether the child was subject to placement change at each of 3 time points – “Stable”, “Unstable” and “Unstable-Stable” groups	CBCL	Fair	“Stable” placement group demonstrated linear improvement in behavioural outcomes over the length of the study. Children in the “Unstable” group also displayed improvement in all domains except Hyperactivity. Children in the Unstable-Stable group demonstrated improvement only when their placement was unstable.
Herrenkohl et al. (2003), USA	Longitudinal	14 Years	N = 212 Age range = 15–18 years Gender = 39% male	Maltreated vs. non-maltreated groups	Semi-structured interview	Fair	Placement instability predicted drug use and school dropout, but not delinquent behaviour (e.g. theft, fraud, drug dealing, property damage, breaking and entering or public order offences)
Ryan and Testa (2005), USA	Retrospective Cohort Study	2 Years	N = 4085 Age = 16 years Gender = 49% male	N/A	Case Files	Fair	Study explored delinquent behaviour, recorded as any violent (e.g. murder, rape, robbery), property (e.g. burglary, arson) or “nonindex” crime (e.g. drug abuse, vandalism). Placement instability predicted delinquent behaviour in males but not females. Males with three or more placements were 1.54 times more likely than those with one placement to have a delinquency petition, and 2.13 times more likely with four placements.
Lewis et al. (2007), USA	Cross-sectional	N/A	N = 102 Age range = 5–6 years Gender = 42% male	Stable vs. unstable placement groups. Comparison group not in care	CBCL	Fair	Unstable group demonstrated significantly more behavioural and inhibitory control difficulties vs. other more stable placement group and comparison group.
Rubin et al. (2007), USA	Prospective cohort study	36 months	N = 729 Age range = 0–15 years Gender = 46% male	3 groups – early stability (stable placement within 45 days), late stability (stable placement beyond 45 days), unstable throughout	CBCL, temperament scores	Good	Placement stability was a significant predictor of behavioural wellbeing at 18 month follow up. Children in the early stability group had 36–63% better CBCL scores at 18 months in comparison to the unstable group, depending on severity of behavioural difficulty at baseline.
Tarren-Sweeney (2008), Australia	Cross-sectional	N/A	N = 347 Age range = 4–11 years Gender = 51% male	N/A	ACC, CBCL	Good	Placement instability was a significant predictor of emotional and behavioural needs, but only when two other confounding variables were included in the hierarchical linear regression model (participant age and age of removal from birth family).
Aarons et al. (2010), USA	Prospective cohort study	36 months	N = 500 Age range = 2–15 years Gender = 44% male	N/A	CBCL	Good	Placement Instability predicted externalising behaviours at 18–36 month time period. Internalising behaviours predicted baseline-18 months in males and 18–36 months in females only.
Proctor et al. (2010), USA	Prospective Cohort Study	8 years	N = 279 Age range = 6–14 years Gender = 46.6% male	N/A	CBCL	Good	Improvements in behavioural wellbeing were predicted by placement stability. Placement stability was a more consistent predictor of behavioural wellbeing than social and cognitive ability.
Prentky et al. (2014), USA	Longitudinal	7 years	N = 559 Age range = 3–18 years Gender = 100% male	4 groups depending on number of placements	Case files	Fair	Placement Instability significantly predicted sexual offence persistence, sexually inappropriate and aggressive behaviours.
Villodas et al. (2016), USA	Prospective Cohort Study	8 years	N = 330 Age range = 4–12 years Gender = 47% male	N/A	CBCL, YSR	Good	Placement instability predicted externalising behaviours from carer report only. Placement instability predicted both internalising and externalising behaviours in child self-report
Bederian-Gardner et al. (2018), USA	Cross-sectional	N/A	N = 146 Age range = 16–18 years Gender = 63% male	Foster care vs. non-foster care comparison	LASC, CHIS, ECR-S	Fair	Placement instability predicted PTSD symptoms in the foster care group only. It did not predict symptoms of anxiety or depression unless school instability was also included.
Okpych and Courtney (2018), USA	Cross-sectional	N/A	N = 706 Age range = 16–18 years Gender = 39% male	6 groups based on foster care characteristics	MINI-KID	Good	Placement Instability predicted depression, PTSD and substance misuse disorder symptomatology.

### Study Findings and Methodological Assessment

The findings for each included study are detailed below, grouped into one of four sections based on the focus of the paper. The results of each individual study are reported, with consideration paid to the possible limitations and methodological biases which may have impacted their results. Results within each subsection are synthesised to clarify the overall findings of the literature.

#### Placement Instability and Behavioural Outcomes

Six studies investigated the impact of placement instability on the behaviour of foster care children. Each of these studies explored internalising and externalising behaviours in children. Internalising behaviours are those associated with anxiety and mood disorders e.g. withdrawal, dysphoria. Externalising behaviours include oppositional, challenging and other problem behaviours e.g. impulsivity and aggressiveness (Zilanawala, Sacker & Kelly, 2019).

One published study reported that placement instability had a deleterious impact on both internalising and externalising behavioural outcomes (Newton et al., 2000). Children with five or greater placement changes (n = 98) over a 12-month period had greater internalising and externalising symptomatology than those with less than five changes in the same period (n = 317). However, five or more placement changes in a single year represents far greater instability than many children in foster care experience (Dolan et al., 2013). This subgroup (deemed to have “volatile” placement histories) may not be indicative of the typical care experience, as evidenced by the group’s smaller numbers (23.6% of the overall sample). Consequently, these groups may not be appropriate to explore the impact of instability on outcomes, particularly as there may still be substantial instability within the group labelled “non-volatile” (up to four changes in a single year), the effects of which are not identified.

Lewis et al. (2007) explored oppositional behaviour and inhibitory control (the ability to moderate and control impulses to align with expected behaviour) abilities of 102 5–6 year old children. Children in the multiple placements group demonstrated significantly more externalising behaviour and inhibitory control difficulties than the stable placement and never-placed groups. However, as a cross-sectional study it is not possible to assume causality in this relationship. Analysis of the overall sample also revealed no significant association between placement instability and internalising behaviours. Importantly this study clustered all children with more than one placement change into a multiple placement group, ignoring the potential additive impact that many placement disruptions may have had. Children in this study, although formerly in foster care, were in a stable adoptive home for at least 10 months prior to participation. The stability of this relationship may have influenced their behaviour in ways which limits the ability to generalise to those in foster care. Adopted children have been shown to have been subject to less pre and post-care adverse experiences and to have better developmental and psychological outcomes than foster care children (Jimenez-Morago, Leon & Roman, 2015; Vinnerljung & Hjern, 2011). They are likely to be substantially different from a foster care population, making direct comparisons arguably inappropriate. The group examined in this study was also predominantly female (62%). Recent demographic studies have found that the foster care population is roughly evenly split by gender in both the US and UK (52–56% male, Children’s Bureau, 2018, Department for Education, 2019) and thus this and other studies weighted towards female participants may not be representative of the wider population of children in foster care.

Aarons et al. (2010) measured CBCL score and placement instability at baseline, 18 and 36 month later. Placement instability between baseline and 18 months did not predict externalising behavioural difficulties, although a significant effect was found between 18 and 36 months, with a medium effect size. Placement instability predicted internalising behaviour difficulties from baseline to 18 months for boys only (small effect size) and at 18–36 months for girls only (medium-large effect size). The authors also reported that there was more evidence for behavioural difficulties predicting placement changes than placement changes predicting subsequent behaviour in the sample. In addition, the mean number of placement changes in the sample was low (m = 1.92 between baseline and18 months and 0.28 between 18 and 36 months), which may have made it difficult to discern the effects of instability.

Villodas et al. (2016) undertook a comparable study to that of Aarons et al. (2010) over a substantially longer time period. Participants were sampled every two years between the ages of 4 and 12, one of the longest longitudinal studies in this review. Child self-report data indicated a relationship between instability and both internalising and externalising symptoms. Caregivers reported a significant impact on externalising but not internalising symptoms. However, only 18% of the sample were reported to have unstable placements throughout the study. The subset with unstable placements were also found to be older at age of entry into care, had more placement changes while in care and had sexual abuse as a disproportionately high reason for removal, in comparison to those in the other groups (12.5% with sexual abuse as reason for removal in the unstable group vs. 1% in children who were adopted and 7% of those in stable foster care). These children may therefore be different from those in the other groups in meaningful ways beyond instability of placement.

An additional two studies did not find evidence that placement instability negatively impacted outcomes. Zima et al., (2000) found that, while 69% of the sample had CBCL scores indicative of behavioural problems, with 27% testing within the clinical range, placement instability was not a significant predictor of behavioural difficulties in the sample. However, placement instability is not well defined in the study and it is unclear what changes in circumstance (e.g. school, emergency placements) are included. Furthermore, the authors note that their case note analysis may be flawed due to how referrals were coded. Children who were reunified with their birth parents and who then re-entered care were given a new case number, with previous placements not included and thus under-reported. In addition, the study reports that participants were sampled from a low-income metropolitan area with high levels of out of home placements. The study results may therefore be impacted by the socioeconomic status of the sample participants.

Barber and Delfabbro (2003) sampled 235 children in foster care between the age of 4 and 17. Current placement was noted at baseline, 4 and 8 months later. Behaviour was assessed via an abbreviated version of the CBCL, administered to the child’s social worker. The results indicated that the behaviour of children with placement stability improved throughout the study. Children in the unstable placement group also demonstrated gradual improvement in their behaviour, and the unstable-stable group demonstrated improvement in their behaviour only while placements were unstable. Weak to moderate effect sizes were reported. The short follow-up timeframe means only the effect of short-term instability on outcomes can be discerned. Furthermore, as placement instability was treated as a categorical variable the impact of multiple placement moves between time points was lost.

#### Placement Instability and Behavioural Outcomes: Summary

Overall, these studies reveal inconsistencies in the literature regarding the impact of placement instability on behavioural outcomes. Results were consistent for externalising behaviours, with a significant relationship reported in four of the six studies above (Aarons et al., 2010; Lewis et al., 2007; Newton et al., 2000; Villodas et al., 2016). Results of the relationship with internalising behaviours were less consistent. Newton et al. (2000) reported a significant relationship, while Villodas found this relationship with self-report data only, and Aarons et al. (2010) demonstrated a differential impact between males and females. The other two studies did not find a significant relationship between instability and behavioural outcomes, although the shorter follow-up period in the Barber et al. study and broader definition of placement instability in the Zima et al. study make these studies harder to compare with the others (Barber & Delfabbro, 2003, Zima et al., 2000). While an advantage of all these studies is that they use the same CBCL outcome measure, they all vary considerably in how instability was measured. Some recorded this metric as a continuous variable and others constructed categorical variables using various cut-offs, making it difficult to draw any firm conclusions.

#### Placement Instability and Delinquency, Offending Behaviours and Criminality

Three studies explored the consequences of placement instability on propensity rates for a range of violent and non-violent criminality and/or other status offences (Prentky et al., 2014; Ryan & Testa, 2005). Results from these studies are inconsistent regarding the relationship between placement instability and Delinquency, Offending Behaviours and Criminality.

Prentky et al. (2014) investigated the impact of placement instability as a risk factor for inappropriate sexual behaviours in a sample of male foster care children. All participants had a previous sanction for sexually inappropriate or aggressive behaviour. Placement instability was recorded at time one, with a range of 1–47 placements recorded (mean = 10). Participants were subsequently placed in one of four quartile groups based on number of placements to which they had been subject. Criminal records were evaluated for sexual re-offending behaviour at a seven year follow up. Results demonstrated that sexual offence persistence, sexually inappropriate and aggressive behaviours were significantly predicted by instability. In addition to the relatively high rate of placement instability in the sample, these young people were previously known to social services due to their sexual offence history and this may have impacted placement decisions.

Ryan and Testa (2005) also assessed the impact of placement instability on behaviour via analysis of delinquency rates of 16-year-old children in the US foster care system via retrospective analysis of case file information. Placement instability significantly predicted delinquency rates in male children in foster care, but not females. The study is, however, solely reliant on records to assess this relationship and the authors note that it may therefore be subject to incomplete and inaccurate information. Furthermore, while delinquency may be the result of behavioural difficulties, there may be other factors which affect these rates in vulnerable children. Factors such as cognitive ability, level of caregiver involvement and quality of peer relationships have also previously been implicated in delinquency rates (Forsyth et al., 2018).

In Herrenkohl et al. (2003), children in foster care and a non-foster care comparison group were asked to self-report on their involvement in one of 39 delinquent and/or criminal behaviours within the preceding year. Foster care placement instability predicted subsequent drug use and school dropout, but did not predict various other factors related to delinquency (selling drugs/sex, theft, fraud), alcohol use or other status offences e.g. truancy. However, the participation rate of 46.39% (212/457) indicates a large number of children who could participate, did not agree to participate. It seems likely that those who committed the most serious offences would be least likely to participate, especially given that the study required self-reporting, which would have placed a significant emotional burden on them by acknowledging their offences.

#### Placement Instability and Delinquency, Offending Behaviours and Criminality: Summary

Placement instability appears to have an effect on rates of delinquency and offending behaviours in children in foster care, although the evidence is not wholly consistent on the types of offending behaviours. Herrenkohl et al. (2003) reported that instability was not associated with more serious offences such as theft, fraud and assault, and Prentky et al. (2014) demonstrated a link with sexual offending.

#### Placement Stability and Behavioural Wellbeing

Two studies in the review focused on how children in stable placements may demonstrate improved behavioural outcomes i.e. a lack of identified behavioural difficulties over time. These studies labelled this “behavioural well-being” or “behavioural adjustment”. Rubin et al. (2007) used propensity score matching to indicate that placement stability was a significant predictor of behavioural wellbeing. Participants were assigned to one of three groups based on degree of placement stability over three time points during the child’s first eighteen months of care. These groups were as follows: Stable placement within forty-five days (“early stable”); stable placement beyond forty-five days (“late stable”); or “unstable” throughout. Children in the early stability group who demonstrated low levels of baseline behavioural difficulties had 36% better scores at 18 month follow up, while those with high levels of baseline behavioural difficulties had 63% better outcomes. The authors note, however, that children in the “early stability” group where likely to be younger at entry into care, have a history of less child welfare interventions and demonstrate more behaviour in the normal range at baseline than the late stable and early stable groups. These factors may be equally important causes of behavioural difficulties between the groups as placement stability. The very broad age range of the participants in the study (birth to fifteen years) impacted the choice of measure of behavioural difficulties. As the CBCL is not normed below the age of two, a separate outcome measure was created for the study (“temperament scores”). This measure was combined with the CBCL to create a single metric for direct comparisons. However, no evidence of the correlation between these metrics is provided, and thus the validity of this metric as a measure of behavioural wellbeing is unestablished.

Proctor et al. (2010) sampled 279 participants following entry into care over an 8-year period. Participants completed the CBCL every two years from when the child was six until the age of fourteen. Placement changes were recorded at each time point, coded with a 1 or 0 depending on whether the caregiver was the same on each previous occasion. Stable or increasing behavioural adjustment trajectories were significantly predicted by placement stability in addition to social competence, cognitive ability and low frequency of physical abuse. Placement stability was the most consistent predictor of adjustment in the sample. However, the study does not account for the possibility of multiple placement changes between time points, only that the child experienced at least one placement change in a two-year period. It is not possible, therefore, to differentiate between children with lower and higher levels of instability. CBCL scores are also dichotomised in the study based on clinical cut-offs and it is difficult to discern whether adjustment is clinically significant or subject to small variance around the cut-off score.

#### Placement Stability and Behavioural Wellbeing: Summary

Children who achieve placement stability, particularly early in their care experience, appear to have better behavioural outcomes than those who find a stable placement later or do not achieve stability. Placement stability was shown in Proctor et al. (2010) to be one of the strongest predictors of positive adjustment in young people, more so than cognitive and social ability. This effect was demonstrated in both a short and long-term study, suggesting these positive benefits may extend throughout childhood.

#### Placement Instability and Mental Health

Three studies included in the review explored placement instability with a primary focus on the mental health of foster care children. It must be noted that all studies highlighted below are cross-sectional in nature and as such it is not possible to infer causality in the relationship.

Tarren-Sweeney (2008) explored the impact of a wide range of placement factors (e.g. stability, carer experience, length of time in care) on foster care children’s mental health. Just over half (56%) of 621 caregivers completed the CBCL and Assessment Checklist for Children (ACC), a measure designed to explore emotional and behavioural needs in children in care (Tarren-Sweeney, 2007). Hierarchical linear regression models indicated that placement instability was only a significant predictor of mental health difficulty when participant age and their age of removal into care were also entered into the model. This suggests that placement instability alone is not a significant risk factor for poor mental health, but rather a concordance of instability with other care-related variables is required to reliably predict mental health outcomes. This study explores a wide range of these variables which may potentially impact outcomes, such as adverse experiences and maltreatment history, cognitive ability, contact with birth family and child and carer demographic factors. However, the study response rate of 56% suggests that the sample may not be representative of the population as a whole, especially as it was noted that non-respondent children entered care earlier, were subject to less maltreatment and more likely to be in a stable placement.

Bederian-Gardner et al. (2018) sampled 146 foster care and 83 non-foster care children. Children in care were enrolled in a government-mandated survey exploring a range of life circumstances (e.g. dependents, homelessness, drug misuse). These young people were also asked to complete three optional measures of mental health difficulty and self-report on their placement and school history. The comparison group was sampled from a school with similar demographic factors as the foster care group to control for variables which may impact the results e.g. socioeconomic status. The results indicated that, while the foster care experience alone did not predict mental health difficulties, residential instability predicted PTSD symptoms in the foster care group. Instability did not, however, predict symptoms of anxiety or depression in this group unless school instability was also included in the analysis. Tentatively this suggests that school stability may be something of a protective factor against anxiety or depression in this group.

Okpych and Courtney (2018) explored placement instability and other foster care characteristics (e.g. age of entry into care, number of years in care) and psychiatric symptomatology, explored via structured diagnostic interview, using the Mini Internal Neuropsychiatric Interview for Children and Adolescents 6.0 (MINI-KID, Sheenan et al., 1998). This measure screens for diagnostic criteria suggestive of possible depression, manic or hypomanic episodes, PTSD and externalising disorders. Participants were grouped via latent mixture modelling into one of 6 classes based on age of entry into care and maltreatment history. Around half of the sample had scores indicating possible mental health disorders. Higher Placement instability subsequently predicted greater likelihood of participants screening positive for depression, PTSD and substance misuse disorder.

#### Placement Instability and Mental Health: Summary

The relationship between placement instability and mental health outcomes has been difficult to identify independently of other concordant factors. While instability was a predictor of PTSD symptomatology in both Bederian-Gardner et al. (2018) and Okpych and Courtney (2018), these studies differed in their findings regarding its impact on mood. The findings in Tarren-Sweeney (2008) highlight that there are potentially a range of placement-related factors which contribute to mental health difficulties. Studies such as these need to control for other care-related factors when assessing the influence of instability on outcomes.

## Discussion

Many of the studies evaluated above appear to demonstrate that placement instability does have some effect on emotional and behavioural outcomes. Evidence was inconsistent, however, in regard to the precise outcomes which are impacted by placement instability. The data indicated a stronger relationship between placement instability and externalising symptomatology in comparison to internalising symptoms. However, this relationship may also be influenced by factors such as gender or the sensitivity of the methodology, e.g. self-report vs. carer-report measures. Placement instability also predicted behavioural difficulties such as delinquency, although these were again inconsistent across studies, possibly due to differences in measurement. Results from studies primarily exploring emotional outcomes suggest a link between instability and PTSD symptoms but differ in their reporting of the impact of instability on mood and anxiety disorders. There was also evidence to suggest that stability of placement is associated with better adjustment and improved behavioural outcomes over time.

One of the primary goals of alternative placements is to foster children opportunities for positive attachment experiences and improved stability and safety in an effort to promote development of functional abilities such as emotion regulation. Unfortunately, negative attachment experiences are also a barrier to forming reparative relationships with caregivers and it has been demonstrated that emotional and behavioural difficulties often affect the attachment relationship (Kernreiter et al., 2020). This can result in more frequent placement breakdowns (Rock et al., 2015), which cause further distress for the young person and reinforce negative internal working models about ‘the self’ and how they are viewed by others (Sattler et al., 2018). This nuanced relationship is likely to have a “cascading effect” (Bederian-Gardner et al., 2018), wherein placement breakdown causes distress for the young person that results in greater behavioural difficulties, which in turn affects the type and quality of subsequent placements and increases the likelihood of further instability. This is not to say that placement stability is always low (instability high) for foster care populations, as in indicated by the diverse comparison groups present in the reviewed studies (some determined as having relatively high stability).

### Methodological Issues and Limitations of this Review

The studies reviewed varied considerably in their comparability of the: (1) samples recruited; and (2) the measurement approaches used, making it difficult to form firm conclusions. Equally important, the reviewed studies varied very little in: (1) the countries health and social care systems; (2) cultural support systems; and (3) consideration of the children’s lived experience, making it difficult to reliability extrapolate the findings to the wider foster care population.

The biggest sampling difference between studies stemmed from different definitions of what constitutes a placement change. Some studies included all changes of residency including short-term emergency placements, while others categorised a change based on how long the child was accommodated. This led to high levels of variance between children in some studies (e.g. Prentky et al., 2014) and small amounts in others (e.g. Aarons et al., 2010) which make them difficult to compare directly. Several studies coded placement instability as a categorical variable as a means to group children e.g. into “Stable” vs. “Unstable” subgroups. Each study provided a rationale for choosing their definition, nevertheless the difference between the studies prevents a clear synthesis of the cumulative impact, if any, of placement instability on behavioural and mental health outcomes. Aside from differences in the definition of stability/instability, the impact of age and development is particularly difficult to quantify across the studies reviewed. Several studies recruited child (e.g. Lewis et al., 2007) or adolescent (e.g. Okpych & Courtney, 2018) samples only, while others used a sample that spanned throughout childhood (e.g. Herrenkohl et al., 2003).

In terms of measurement approaches, for many of the variables assessed, the studies were longitudinal in nature, which is typically seen as a design strength. However, children who experience multiple placements will, by definition, have different respondents completing the questionnaires at different time points. These respondents may differ in the how much they focus on the particular symptoms or difficulties presented by the children in their care. Essentially children with unstable placement histories may not have as consistent or reliable data over time as those with stable placements. The majority of studies reviewed have measured behavioural outcomes, rather than emotional and mental health outcomes. Furthermore, most of the studies discussed used carer report measures that are screeners rather than more sophisticated diagnostic tools. For example, eight of the fourteen included studies used the CBCL as a measure of behavioural difficulties. The samples for these eight studies were largely derived from wider cohort studies in the US of young people who come to the attention of mental health services, with the CBCL used as part of routine outcome monitoring. The CBCL is not designed specifically for children in foster care, a population which often differs from the normal population in the severity of cognitive, psychological and social difficulties (e.g. Jacobsen et al., 2020, Pears & Fisher, 2005). Consequently, it may be the case that the measure does not provide an accurate representation of behavioural difficulty in this population. It should be noted, however, that CBCL scores have been shown to correlate highly with ACC scores (a comparable measure designed specifically for a foster care population; Tarren-Sweeney, 2008).

In the studies reviewed, there is a clear absence of the voice of the children involved providing lived experience. This is likely to be a result of the systematic review criteria which excluded purely qualitative publications (e.g. Ellermann, 2007). While this is essential to evaluate the relationship between placement stability and outcomes, it does not consider children’s perceptions of their experience which may not always equate with their behaviour (Cooley et al., 2015). Equally, limiting is the geographic distribution of the studies reviewed. All reviewed studies originated from either the USA (n = 12) or Australia (n = 2) with no other nationalities represented. This is limitation to representativeness, given the inherent differences in social care and corporate parenting systems between countries (Harlow, 2022). There may be particular factors within the systems of these countries that makes placement instability more common or additional variables which contribute to negative outcomes of children in care. Indeed, other countries such as the UK appear to have better levels of placement stability than many of the articles reviewed (McSherry & Fargas-Malet, 2018).

### Research Implications

The current review highlights the difficulty often inherent in research in looked after children and presents an opportunity to highlight the importance of developing more robust research methods for children in care in general, including in relation to placement stability. Firstly, there is a need for an operational definition of placement instability from which to base subsequent research in the area, including what constitutes a placement move (a figure likely influenced by social policy differences between research sites) and the use of categorical versus continuous variables in a study. On the whole, transforming placement stability into a categorical variable (e.g. stable or unstable) reduces the sensitivity and application of the measure, especially given the high numbers of placement moves to which some children are subject. Secondly, there is a clear need for research to originate from a wider range of countries taking into account differences within the care system between nations. Thirdly, there is a clear need to consider a much wider range of care-related factors than just placement stability/instability. Children’s experience of care is often highly varied and subject to a wide range of placement and care related factors which will likely contribute to emotional and behavioural outcomes. Research in future should attempt to account and control for additional factors such as age of entry into care, carer characteristics and pre-care experiences when assessing the significance of the influence of placement instability on outcomes. In one example, Berger et al. (2009) explored behavioural outcomes in children in foster care while controlling multiple variables such as child and caregiver age, socioeconomic status, educational attainment, maternal mental health and maltreatment history. Without controlling for the above factors, out of home placement significantly predicted internalising and externalising behaviour. When controlling for other variables, this effect was no longer present in the case of externalising behaviour. Clearly the choice of control variables has a significant impact of the conclusions drawn. Finally, studies that can assess causal influences are particularly important. Current research regarding the impact of instability on mental health outcomes is cross-sectional and would benefit from a robust longitudinal study to fully explore the impact over time. Importantly, Aarons et al. (2010) found that behavioural difficulties predicted later placement instability better than placement instability predicted behavioural difficulties. This lends credence to the idea that the relationship between placement instability and emotional and behavioural outcomes is bidirectional in nature, but this needs to be more consistently researched (see Konijn et al., 2019, for a review).

Children in foster care are subject to a wide range of placement and trauma-related factors which may impact both emotional and behavioural outcomes and placement stability (e.g. Rayburn et al., 2016). Most of the studies reviewed made efforts to control for some of these variables, such as age, age of entry into care and maltreatment history. However, it is difficult to control for all of the important variables in a single study. Importantly, the study that controlled for the most variables reported no relationship between placement instability and emotional and behavioural outcomes unless age of the child and age of removal into care were both included in the regression model (Tarren-Sweeney, 2008). While two of the studies reviewed examined the positive effects of placement stability on behavioural wellbeing, little consideration has been given to potential moderators or mediators of this relationship. Clearly more studies need to consider care-related factors such as placement type (Barth et al., 2007), foster carer characteristics (Barber et al., 2001) and quality of parenting (Chodura et al., 2021) alongside placement stability. Similarly, more studies should consider the potential protective factors have been largely ignored. Research has shown for example, that when the care experience is of a high quality then children’s wellbeing can reflect this (e.g. Shpiegel et al., 2022) and indeed when other care-related factors are favourable this may have an ameliorating effect even if stability cannot be achieved (Tarren-Sweeney, 2008).

### Clinical Implications

The literature related to foster care children often emphasises the importance of placement stability as a protective factor against negative emotional and behavioural outcomes (e.g. McSherry & Fargas-Malet, 2018). The current review identified multiple studies which support this suggestion, although there was no consensus and several studies found either no relationship or a relationship for only some outcome variables. Taken as a whole, there appears to be a trend towards better outcomes for children with more stable placement experiences. While services should continue to focus on ensuring that children in care are given every opportunity to establish a stable and supportive placement, assessment of broader factors like the quality of peer relationships (Forsyth et al., 2018) and caregiver factors (Miller et al., 2019) should also be considered relative to the behavioural difficulties experienced (e.g. delinquency, or anxiety and depression, respectively). The overall goal should still be to support children to develop a secure base and a sense of relational permanence, including through development of a stable placement experience. Providing optimal care for this population requires a holistic approach to all aspects of the care experience (Harder et al., 2020), with placement stability appearing to be one important consideration.
